# *BRCA1* and *BRCA2* founder mutations account for 78% of germline carriers among hereditary breast cancer families in Chile

**DOI:** 10.18632/oncotarget.18815

**Published:** 2017-06-29

**Authors:** Carolina Alvarez, Teresa Tapia, Elisa Perez-Moreno, Patricia Gajardo-Meneses, Catalina Ruiz, Mabel Rios, Claudio Missarelli, Mariela Silva, Adolfo Cruz, Luis Matamala, Luis Carvajal-Carmona, Mauricio Camus, Pilar Carvallo

**Affiliations:** ^1^ Department of Cell and Molecular Biology, Faculty of Biological Sciences, Pontificia Universidad Católica de Chile, Santiago, Chile; ^2^ Centro de Cáncer, Faculty of Medicine, Pontificia Universidad Católica de Chile, Santiago, Chile; ^3^ Unidad de Patología Mamaria, Hospital Base de Valdivia, Valdivia, Chile; ^4^ Unidad de Patología Mamaria, Hospital Barros Luco Trudeau, Santiago, Chile; ^5^ Unidad de Patología Mamaria, Hospital Regional de Antofagasta, Antofagasta, Chile; ^6^ Genome Center and Department of Biochemistry and Molecular Medicine, School of Medicine, University of California, Davis, California, USA

**Keywords:** BRCA1, BRCA2, founder mutation, breast cancer, Chile

## Abstract

Identifying founder mutations in *BRCA1* and *BRCA2* in specific populations constitute a valuable opportunity for genetic screening. Several studies from different populations have reported recurrent and/or founder mutations representing a relevant proportion of *BRCA* mutation carriers. In Latin America, only few founder mutations have been described. We screened 453 Chilean patients with hereditary breast cancer for mutations in *BRCA1* and *BRCA2*. For recurrent mutations, we genotyped 11 microsatellite markers in *BRCA1* and *BRCA2* in order to determine a founder effect through haplotype analysis. We found a total of 25 mutations (6 novel) in 71 index patients among which, nine are present exclusively in Chilean patients. Our analysis revealed the presence of nine founder mutations, 4 in *BRCA1* and 5 in *BRCA2*, shared by 2 to 10 unrelated families and spread in different regions of Chile. Our panel contains the highest amount of founder mutations until today and represents the highest percentage (78%) of *BRCA1* and *BRCA2* mutation carriers. We suggest that the dramatic reduction of Amerindian population due to smallpox and wars with Spanish conquerors, a scarce population increase during 300 years, and the geographic position of Chile constituted a favorable scenario to establish founder genetic markers in our population.

## INTRODUCTION

It is well known that an early detection of breast cancer is highly relevant in relation to patient survival, for this reason a genetic analysis revealing a high risk to breast cancer for healthy women constitutes valuable information. Twenty years ago two genes, *BRCA1* (MIM: 113705) and *BRCA2* (MIM: 600185), which mutations confer a high risk to breast cancer were identified in families having four or more relatives with breast or ovarian cancer [1, 2]. Since then, the screening of *BRCA1* and *BRCA2* has been performed in multiple populations across the world revealing that 10 to 50% of breast cancer patients with family history of breast and/or ovarian cancer carry a *BRCA1* or *BRCA2* mutation [3]. In Chile, we and others have carried out mutation screening of *BRCA1* and *BRCA2* in hereditary breast cancer patients, leading into mutation frequencies ranging from 15% [4] to 20% [5]. Among Latin American countries, eight similar studies have been published up today: México [6], Colombia [7], Brazil [8], Argentina [9], Uruguay [10], Venezuela [11] and Chile [4, 5]. Among those studies the frequency of mutation carriers in Latin American women with hereditary breast cancer varies from 15% to 25%.

The presence of founder mutations in *BRCA1* and *BRCA2* in specific ethnic groups or populations constitutes a valuable opportunity for a genetic screening. The most well-known example is the Ashkenazi Jewish population [12], in which three founder mutations combined have a population frequency of 2% and represent 60% of breast cancer families with a BRCA gene mutation. A second well-known example is the Icelandic population in which one founder mutation in *BRCA2* 999del5 has a population carrier frequency of 0.4%, and account for 64% of breast cancer families [13, 14, 15]. In the last 15 years several studies have described recurrent mutations in breast cancer patients, some of which have documented a single origin (founder effect) of such recurrent mutation through haplotype analyses. In this relation, it is necessary to highlight that a recurrent mutation is not necessary founder, since some recurrent mutations may correspond to hotspots, arising independently in a population. To be founder, a minimum common haplotype must be identified among carriers of a certain recurrent mutation. In Poland [16] three founder mutations account for up to 86% of all families with a *BRCA1* or *BRCA2* mutation. In Slovenia five founder mutations account for 69% of all BRCA families [17]. Several other studies from Europe, Asia and North America have reported recurrent and/or founder mutations that represent a relevant proportion of *BRCA1* and *BRCA2* mutation carriers [18]. In Latin America, there are few different mutations with a demonstrated founder effect described in different populations: Mexico (*BRCA1* ex9–12del), Brazil (*BRCA1* 5382insC), Colombia (*BRCA1* 3450del4, A1708E, and *BRCA2* 3034del4) and US Hispanics (*BRCA1* 185delAG, IVS5+1G>A, S955X, and R1443X) [19, 20]. In addition to these founder mutations, other recurrent mutations have been described in Latin American populations which founder effect through haplotype analyses still has not been tested.

In Chile, our group has screened *BRCA1* and *BRCA2* genes in 336 patients with a family history of breast cancer and 117 patients unselected for family history, recruited throughout the country. The results of this study led us to define a panel of 9 founder mutations among Chilean patients representing 77.5% of total BRCA mutation carriers.

## RESULTS

### Novel and recurrent mutations in *BRCA1* and *BRCA2*

We identified 25 different mutations (pathogenic variants, class 5), of which 6 are novel and 19 have been previously reported (Table 1). These mutations are present in 71 index patients, of which 32 carried a mutation in *BRCA1* (7.06%) and 39 (8.61%) in *BRCA2* leading to a combined mutation prevalence of 15.7% (71/453). It is noteworthy that considering only cases with family history this percentage is higher, being 17.9 %. Nine of the 25 mutations described in this work have been found exclusively in Chilean patients [4, 5, 21] (and this study), two of them being recurrent in our samples (c.1504_1507delTTAA and c.3817C>T, both in *BRCA1*) (Table 1). We have to remark that mutation p.Asp2723Gly has been previously described (BIC) as a variant of uncertain significance in several populations and in 2008, a functional analysis made by Farrugia et al. [22] defined that this aminoacid change was detrimental for *BRCA2* function in DNA repair and centrosome duplication. Since then this mutation has been defined as pathogenic. Mutation c.211A>G in *BRCA1* (previously named as R71G) is located in the before last nucleotide of exon 5, eliminating the donor splice site in this exon and revealing a cryptic splice site 22 nucleotides upstream. The deletion of 22 nucleotides in the mRNA creates a stop codon in position 64 [23]. We corrected the protein nomination for this mutation from the previous p.Arg71Gly to p.Cys64Ter.

**Table 1:** Mutations in *BRCA1* and *BRCA2* in Chilean breast cancer patients

Gene	Number of families	Exon	Systematic nomenclature (HGVS)		Previously named as		Population(s) Reported
			cDNA	Protein	cDNA	Protein	
*BRCA1*	1	2	c.68_69delAG	p.Glu23Valfs*17	185delAG	Stop 39	Ashkenazi-Jewish
	1	5	c.181T>G	p.Cys61Gly	300T>G	C61G	Europe
	1	5	c.187_188insA	p.Leu63Tyrfs*3	308insA	Stop 65	Chile^1^
	1	5	c.211A>G	p.Cys64Ter	330A>G	R71G	Global
	1	7	c.303T>A	p.Tyr101Ter	-	-	NO
	2	11	**c.1504_1507delTTAA**	p.Leu502Serfs*29	-	-	NO
	1	11	c.2486_2487delTT	p.Phe829Ter	2605_2606delTT	F829X	Chile^2^
	9	11	**c.3331_3334delCAAG**	p.Gln1111Asnfs*5	**3450del4**	**Stop1115**	Global
	7	11	**c.3759dupT**	**p.Lys1254Ter**	**3878insT**	**Stop 1254**	Global
	4	11	**c.3817C>T**	**p.Gln1273Ter**	**3936C>T**	**Q1273X**	Chile^1^
	1	11	c.3858_3861delTGAG	p.Ser1286Argfs*20	3977del4	Stop 1305	China, Chile^2^
	1	11	c.3968_3971delAAAT	p. Gln1323Argfs*11	-	-	NO
	1	11	c.4065_4068delTCAA	p. Asn1355Lysfs*10	4184del4	Stop 1364	Global
	1	20	c.5266dupC	p.Gln1756Profs*74	5382insC	Stop 1829	Ashkenazi-Jewish
*BRCA2*	5	3	**c.145G>T**	**p.Glu49Ter**	**373G>T**	**E49X**	Globally
	10	11	**c.4740_4742dupTG**	p.Glu1581Valfs*37	**4970insTG**	**Stop 1617**	Chile^1,2^ Argentina^3^
	9	11	**c.5146_5149delTATG**	p.Tyr1716Lysfs*8	**5373delGTAT**	**Stop 1724**	Spain and Chile^1,2^
	1	11	c.5946delT	p.Ser1982Argfs*22	6174delT	Stop 2003	Ashkenazi-Jewish
	1	11	c.6629_6630delAA	p.Glu2210Glyfs*14	6857delAA	Stop 2223	Spain and Chile^1^
	1	14	c.7397dupC	p.Ala2466Alafs*8	-	-	NO
	1	18	c.8168A>G	p.Asp2723Gly	8396A>G	D2723G	Global
	1	18	c.8223_8224dup11	p.Asn2742Leufs*	-	-	NO
	1	22	c.8941G>T	p.Glu2981Ter	-	-	NO
	6	23	**c.8987T>A**	**p.Leu2996Ter**	-	-	Once in ClinVar origin not provided
	3	25	**c.9382C>T**	**p.Arg3128Ter**	**9610C>T**	**R3128X**	Global

^1^Gallardo et al. 2006, ^2^González-Hormazabal et al. 2011, ^3^Solano et al., 2016. NO: not reported previously, first mentioned in this manuscript. GeneBank Accession Numbers: *BRCA1:* L78833 and BRCA2: AY436640.

Considering all mutations (*n* = 25) 9 were recurrent in our sample set (Table 1, highlighted in bold). This group of nine mutations is present in 77.5% (55/71) of total BRCA carriers, becoming an excellent panel for cost-effective *BRCA1* and *BRCA2* mutational screening in Chilean patients. The most frequent of these nine recurrent mutation is c.4740_4741dupTG in *BRCA2* (Table 1). This mutation was first described in Chilean patients by our group [5] and later found by two groups in unrelated Chilean [21], and Argentinian patients [24]. Two other frequent recurrent mutations (*BRCA1* c.3331_3334delCAAG and BRCA2 c.5146_5149delTATG) were found in 9 families each.

### Common haplotypes found for recurrent *BRCA1* and *BRCA2* mutations

**Figure 1:**
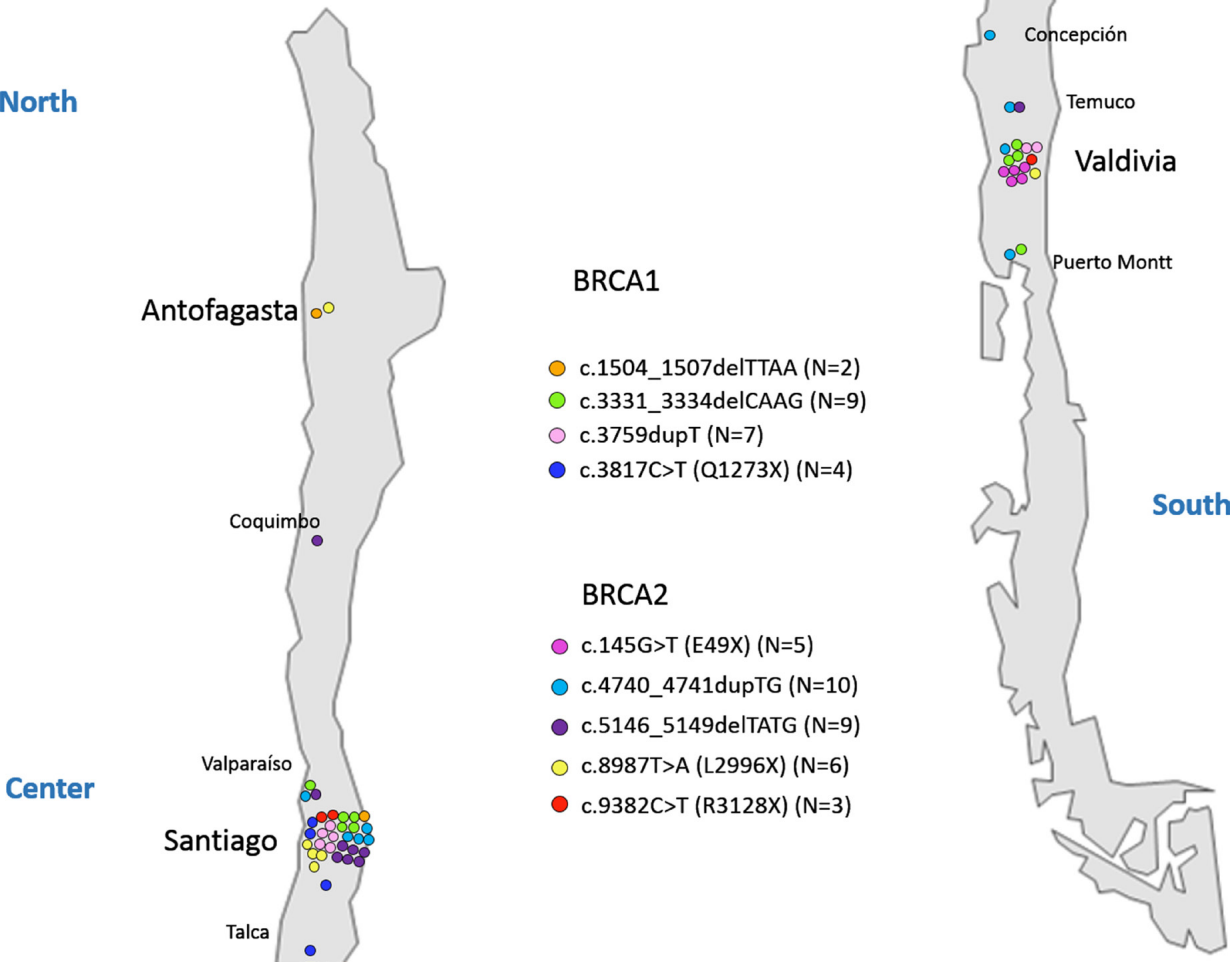
Geographical distribution of recurrent mutations A map of the Chilean territory indicating cities where patients were recruited is shown. Each mutation is represented with specific color, and each carrier family is indicated by a colored circle.

In order to determine a probable founder effect for the 9 recurrent mutations we analyzed 11 STR markers (6 for *BRCA1* and 5 for *BRCA2*) in 53 index patients and established the corresponding haplotypes. Patients sharing the same mutation belong to apparently unrelated families residing in different regions along Chile as it is shown in Figure 1.

**Figure 2:**
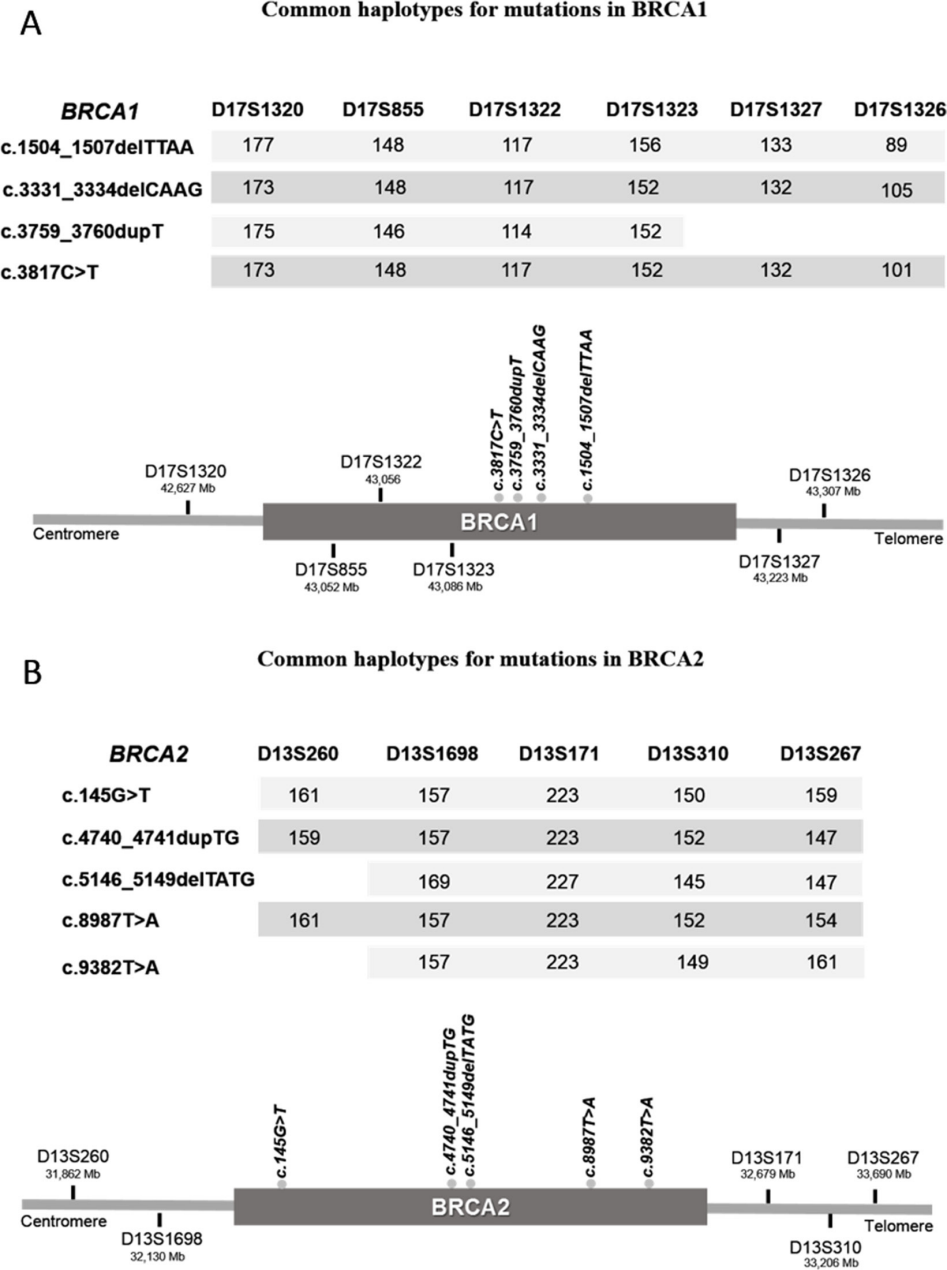
Minimum common haplotypes identified for each recurrent mutation in *BRCA1* (**A**) and *BRCA2* (**B**). Values under each STR marker indicate the size of the shared alleles. Scheme (bottom image) represents the localization of all markers and mutations in relation to each gene.

All microsatellites for *BRCA1* and *BRCA2* were informative (Figure 2). For three mutations in *BRCA1,* c.1504_1507delTTAA (2 families), c.3331_3334delCAAG (9 families) and c.3817C>T (4 families), the core haplotype (identical haplotype at loci) was shaped with the six microsatellites markers (Figure 2A) revealing no recombinants in a region of 0.68 Mb. For the fourth mutation, c.3759dupT, the core haplotype was shaped with four markers (Figure 2A) covering a region of 0.46 Mb. In this case one sample is a recombinant for D17S1326 and two samples for markers D17S1327 and D17S1326. On the other hand, for three mutations in *BRCA2,* c.145G>T (3 families), c.4740_4741dupTG (6 families) and c.8987T>A (6 families), the core haplotype was shaped with the five microsatellites markers (D13S260, D13S1698, D13S171, D13S310 and D13S267) revealing no recombinants in a region of 1.82 Mb (Figure 2B). Conversely, for mutations c.5146_5149delTATG (8 families) and c.9382T>A (3 families) we found one recombinant for D13S260 in our samples. For both mutations the minimum common haplotype covers 1.56Mb (Figure 2B). We were able to determine a common haplotype for all the analyzed mutations.

### Polymorphisms and unclassified variants in *BRCA1* and *BRCA2*

We found a total of 110 coding and non-coding variants, 15 not previously reported in the literature or public data bases. Among the 95 reported variants, 62 correspond to polymorphisms (allelic frequencies ≥ 1%, not pathogenic, class 1) reported in at least one population in the 1000 Genomes database. In addition we detected 33 rare variants (Supplementary Table 1), either with allelic frequencies < 1% in all populations reported (1000 Genomes or dbSNP) or allelic frequencies not reported. The classification of the 33 rare variants (Supplementary Table 1) was concluded after revising three databases (BIC, ClinVar, BRCA Share) and functional assays in published papers. Nineteen of these variants have been classified as “benign” or “likely benign” (also neutral/likely neutral) in ClinVar and BRCA Share databases (Supplementary Table 1). Seven variants are reported as “unclassified variant” or “variant with uncertain significance” (Supplementary Table 1: ClinVar, BRCA Share). The remaining 7 variants have a controversial classification, being reported as benign/likely benign (neutral/likely neutral) by one database and uncertain by another. Among the 14 variants reported previously, at least once in data bases as “uncertain”, three have been tested in functional assays concluding a neutral or non-pathogenic effect, indicated under conclusion in Supplementary Table 1. In summary, according to ENIGMA recommendations and considering reported *in silico* analyses, functional assays, and co-occurrence with a *BRCA1/2* pathogenic variant, there are 17 not pathogenic and 6 likely not pathogenic variants. For 10 rare variants there was insufficient information so were interpreted as “uncertain” (Supplementary Table 1).

In relation to novel variants in this study a total of 11 are described in Table 2. Among these, 6 are coding variants and 5 non-coding. In the coding region only two novel synonymous variants were found in our study. These variants are predicted to produce a weak or null effect in splicing by the three utilized tools. On the other hand among these, 2 variants, p.Pro34 = co-occur with a mutation in *BRCA1*, c. 211A>G. These results suggest that these variants should be classified as neutral or non-pathogenic.

**Table 2:** Novel variants in BRCA1 and BRCA2 in Chilean patients

Gene	Exon/Intron	Hgvs Nomenclature	Protein Effect	Number of Breast Cancer Patients	Co-ocurrence with BRCA Pathogenic Variant	*In silico* Analysis
	**SYNONIMOUS VARIANTS**					
*BRCA1*	3	c.102T>G	p.Pro34=	1/306	YES	Null effect (a,b,g)
*BRCA1*	20	c.5211A>G	p.Arg1737=	1/306	NO	Null effect (a,b,g)
	**NON-SYNONIMOUS VARIANTS**					
*BRCA1*	11	c.1961A>C	p.Lys654Thr	1/306	NO	Damaging (d); Null effect (a,c,e,f)
*BRCA1*	16	c.4746C>G	p.Asp1582Glu	1/306	NO	Null effect (a,c,d,e,f)
*BRCA2*	11	c.2542A>C	p.Lys848Gln	1/306	NO	Damaging (e,f);Null effect (a,c,d)
	**STOP CODON VARIANT**					
*BRCA2*	27	c.10225C>T	p.Gln3409Ter	1/306	NO	Damaging (a)
	**NON-CODING VARIANTS**					
*BRCA1*	I11	c.4097–164T>C	NO	1/306	NO	-
*BRCA1*	I20	c.5278–21C>T	NO	2/306	NO	-
*BRCA2*	I3	c.316+73A>G	NO	1/306	NO	-
*BRCA2*	I3	c.316+135G>A	NO	1/306	NO	-
*BRCA2*	I22	c.8954–74T>C	NO	1/306	NO	-

a) HCI, Breast Cancer Genes Prior Probabilities, b) VEP, Variant Effect Predictor, c) Align-GVGD, d) SIFT, e) PROVEAN, f) PolyPhen-2, g) HSF, Human Splice Finder.

Three variants are novel non-synonymous (Table 2), and none of them co-occur with a BRCA mutation. Determining the functional effect of a missense variant is a relevant step to interpret their possible pathogenic role in breast cancer. The analysis tools give contradictory predictions for 2/3 variants, so these should be classified as uncertain, until a functional effect can be assessed. Variants c.4746C>G has a null effect predicted by all tools.

Finally, we found one novel truncating variant in exon 27 of *BRCA2* (Table 2: p.Gln3409Ter) downstream of p.Lys3326Ter, which is frequently described as neutral (ClinVar). For this reason we assumed that the new variant should also be neutral as recommended by ENIGMA. Non-coding novel variants are deep in the introns and according to ENIGMA recommendations and ACMG guidelines they should be considered as non-pathogenic. None of these novel variants co-occur with a pathogenic mutation.

## DISCUSSION

We described the screening of *BRCA1* and *BRCA2* in a Chilean cohort of 453 patients. As shown in Table 3 the higher percentage of patients with a mutation in *BRCA1/2* is among families with a male breast cancer (40%, only in *BRCA2*), followed by the group having breast and ovarian cancer cases (37.7%) being higher for *BRCA1*. In relation to patients with no family history the highest percentage of mutation carriers is among bilateral breast cancer patients. It is noteworthy that in the group of younger patients < 40 y 6/7 patients have a mutation in *BRCA1*. In summary the distribution of BRCA mutation carriers is not remarkably different to what has been already described in other breast cancer cohorts. In relation to clinico-pathological characteristics the only relevant data is that among BRCA1 mutation carriers a 58% are triple negative (ER-, PR- and HER2-), as it has been also described by other groups. Analyzing the group of triple negative patients 70% has a mutation in BRCA1.

**Table 3:** Distribution of BRCA1 and BRCA2 mutation carriers according to selection criteria

Patients classification	Total Patients	With mutation		Without mutation, *n* (%)
		*BRCA1, n* (%)	*BRCA2, n* (%)	
***With family history***				
Three or more relatives with breast cancer > 45 y	73	1 (1.4%)	5 (6.8%)	67 (91.8%)
Two relatives with breast cancer, one < 45 y	200	11 (5.5%)	19 (9.5%)	170 (85.0%)
Two or more relatives, one with ovarian cancer	53	12 (22.6%)	8 (15.1%)	33 (62.3%)
Two or more relatives, one male breast cancer	10	0 (0%)	4 (40%)	6 (60.0%)
Total	336	24 (7.1%)	36 (10.7%)	276 (82.2%)
***No family history***				
Diagnosed < 40 y	84	6 (7.1%)	1 (1.2%)	77 (91.7%)
Bilateral breast cancer	27	2 (7.4%)	2 (7.4%)	23 (85.2%)
Breast and ovarian cancer	6	0 (0.0%)	0 (0.0%)	6 (100%)
Total	117	8 (6.8%)	3 (2.6%)	106 (90.6%))

The screening of 453 high risk breast cancer patients from different regions of Chile revealed the presence of nine founder mutations, 4 in *BRCA1* and 5 in *BRCA2*. These mutations are shared by 2 to 10 unrelated families spread in different regions of Chile, and correspond to 77.5% (55/71) of mutation carriers. All unrelated patients having the same mutation share a minimum haplotype revealing a common ancestor for each mutation. In several populations, as it is mentioned in the introduction, founder or recurrent mutations have been described; however the Chilean panel is until today the one containing the highest amount of founder mutations and also representing the highest percentage (77.5%) of *BRCA1* and *BRCA2* mutation carriers, in a single population. These results reveal an excellent low cost panel to screen *BRCA1* and *BRCA2* carriers in Chile, among breast cancer patients including cases apparently sporadic.

It is important to highlight that the three Ashkenazi-Jewish founder mutations are not frequently observed in our population as occurs in Argentina and Brazil. Considering the two large studies in Chile that include almost 800 patients [21] (and this study) three families carry the *BRCA1* c.68_69delAG (formerly known as 185delAG), two carry *BRCA2* c.5946delT (also known as 6174delT), and one present *BRCA1* c.5266dupC (formerly known as 5382insC), representing less than 7% of Chilean mutation carriers, while in Argentina is close to 35% [24]. In the case of our group of families among 13 Ashkenazi Jewish only 2 present a mutation. Family with c.5266dupC is not from Ashkenazi Jewish origin. These results suggest that the Jewish community in Chile have a different ethnic origin compared to those in Brazil and Argentina, or that the prevalence of these mutations among the founders of this community in our country was lower than that in other countries from the region.

In Latin America, there are a few mutations with a demonstrated founder effect described in different populations: Mexico (*BRCA1* ex9–12del), Brazil (*BRCA1* 5382insC), Colombia (*BRCA1* 3450del4, A1708E, and *BRCA2* 3034del4) and US Hispanics (*BRCA1* 185delAG, IVS5+1G>A, S955X, 2552delC and R1443X) [20, 25, 26]. Other recurrent mutations have been described in Latin American populations which founder effect has not yet been tested. The majority of mutations in *BRCA1* or *BRCA2* found in Latin-American breast cancer patients are private for each population, with very few mutations shared between countries [27]. Recently a panel of *BRCA1* and *BRCA2*, HISPANEL [28] has been constructed with diverse mutations from Hispanic breast cancer women from USA, based on the information in manuscripts describing mutations in *BRCA1* and *BRCA2* from Latin American countries and data bases. The HISPANEL, including close to 100 recurrent mutations, has been applied in breast cancer patients from Mexico, Colombia, Peru and Brazil, with diverse results [29–32], due to the diversity of *BRCA1* and *BRCA2* mutations among different Latin American populations, and the variable criteria to select the patients to be analyzed. Indeed our study as well as another study in Chilean patients [21], have shown that only 6 Chilean mutations are present in the HISPANEL, among which only three are Chilean founder mutations, representing only 25% of mutation carriers. This analysis confirms that the HISPANEL is not reliable for screening *BRCA1/2* mutations in our breast cancer patients. In addition we found very few mutations shared with other Latin-American populations revealing that most mutations where introduced in the continent by independent events.

In relation to the 9 founder mutations described in this study c.1504_1507delTTAA and c.3817C>T in *BRCA1* are not described elsewhere consisting in private mutations from Chile. In addition c.4740_4741dupTG in *BRCA2* was also detected in two families from Argentina, and c.145G>T in *BRCA2,* was described in one Argentinian family. Due to the frequent migration events between these two countries this finding is not rare. It is noteworthy that mutation c.145G>T in *BRCA2* was detected exclusively in patients recruited at Valdivia (South of Chile), probably representing a specific settlement of colonists (Figure 1). In the contrary the other 8 founder mutations are present in different cities along the Chilean territory (Figure 1).

An interesting case is the mutation in *BRCA1,* c.3331_3334delCAAG, that is also founder in Colombia, Spain and Portugal (Tuazon A, manuscript in preparation), and also recurrent in 4 Brazilian families [33]. This mutation has been globally described among Caucasian population from Europe, Australia and America (BIC). In concordance with our history of colonization *BRCA1* founder mutations c.3331_3334delCAAG and c.3759dupT, as well as BRCA2 founder mutations c.145G>T and c.5146_5149delTATG have been described in Spain. This latter mutation, c.5146_5149delTATG in *BRCA2*, was first reported in families from the regions of Castilla-León [34] and Aragon [35] in Spain and later stablished as a Spanish founder mutation [36] originated at Castilla-León.

The finding of 9 founder mutations among Chilean breast cancer families is a consequence of history of colonization, and how the population of Chile was settled. Several historical records mention that the indigenous population in Chile at the time of the arrival of Spaniards in 1541 was around 1 million, but it was dramatically reduced as a consequence of battles with Conquerors, dying mainly male indigenous, as well as diseases (Instituto Nacional de Estadísticas, INE 2009). In addition mostly male Spaniards came to Chile during the first three centuries, with a very scarce population increase; therefore the genetic bases of the Chilean population come from mating between Amerindian women and Spanish men [37] (and INE, 2009). These historic issues have been revealed in a previous study from our group, demonstrating that the Chilean population is composed in 84% by Amerindian mitochondrial DNA (maternal lineage) and 32% of Amerindian Y chromosome (paternal lineage) [38]. Even though at the end of the XIX century considerable German and French migrations occurred, and others thereafter, the genetic composition of the Chilean population today still reflects the history of the first three centuries [39]. We could suggest that the dramatic reduction of the Amerindian population in conjunction with the arrival of Spanish conquerors (highly predominant in males), for close to 300 years, was a favorable scenario to establish founder genetic markers in the population. In addition the geographic position of Chile is also beneficial to establish founder genetic markers or mutations since no immigrations came from the Pacific Ocean, few from the north of Chile and very scarce from Europe through Argentina.

## MATERIALS AND METHODS

### Patients

A total of 336 patients with familial breast/ovarian cancer were recruited from 1999 to 2015 using either of the following family history criteria: 1) three or more relatives with breast cancer diagnosed after 45 years old, 2) two relatives with breast cancer at least one diagnosed before age 45, 3) one breast cancer and one ovarian cancer at any age, or 4) one male breast cancer and one female breast cancer. The 117 patients with breast cancer and no family history were selected by 5) being diagnosed at 40 years old or younger, 6) presenting bilateral breast cancer or 7) presenting breast and ovarian cancer. The selected patients were recruited from three Public Hospitals (Antofagasta, Valdivia and Santiago) and one private Institution (Santiago). Two of the public Hospitals assist patients from Regions XV, I, II, and III (Antofagasta Hospital, North of Chile) and IX, XIV, X and XI (Valdivia Hospital, South of Chile), covering 4.5 million Chilean inhabitants (28% of total Chile. Based on Cense 2012). The third Public Hospital (Santiago) assists close to 25% of total patients from the Metropolitan Region. Low and middle income patients attend to these three hospitals.

After informed consent, blood samples were obtained from affected individuals and relatives. This protocol, which adheres to the Helsinki declaration and local regulations, was approved by the Ethics Committees belonging to all the Hospitals where the patients came from. All patients received genetic counselling before and after the genetic test.

### Mutation screening

DNA was isolated using the method described by Lahiri and Nurnberger [39]. PCR amplification covering all coding sequences and intron-exon boundaries, of exons 2 to 24 (*BRCA1*) and 2 to 27 (*BRCA2*) was performed by standard methods. In the first 147 patients, *BRCA1* and *BRCA2* mutations were screened through heteroduplex analysis, Protein Truncation Test (PTT) Single Strand Conformational Polymorphism (SSCP), and Sanger sequencing of selected exons. In 306 patients mutation screening was performed by next-generation sequencing (NGS). Enrichment of BRCA1 and BRCA2 sequences for NGS was performed with BRCA MASTR v2.1 (Multiplicom, Belgium) as follows. Briefly, 5 multiplex reactions were used to amplify all coding exons and intron/exon boundaries of *BRCA1* and *BRCA2* (93 amplicons), followed by sample barcoding. Sequencing was performed in a Junior Roche 454. Analysis of sequencing data was performed with Amplicon Variant Analyzer (AVA) software (Roche) using references sequences for *BRCA1* and *BRCA2* provided by Multiplicom, and filtered to allow the call of all possible variants. The obtained variants were filtered manually using a 30× depth cutoff for each forward and reverse sequences, and allelic variant frequencies over 30%. Allelic frequencies for heterozygous variants were determined between 30 and 70%, and for homozygous variants over 70%. All detected variants were confirmed by Sanger sequencing, and named according to the Human Genome Variation Society (http://www.hgvs.org/mutnomen/) and relative to GenBank reference sequences (*BRCA1*: L78833 and *BRCA2*: AY436640). In addition, all new mutations identified in the 306 patients analyzed by NGS were re-screened in the first group of patients.

### Clinical significance determination for sequence variants

Novel variants were defined as pathogenic (class 5), likely pathogenic (class 4), uncertain significance (VUS; class 3), likely not pathogenic (class 2) and not pathogenic (class 1) following recommendations of the American College of Medical Genetics and Genomics [40] and ENIGMA (ENIGMA consortium, https://enigmaconsortium.org/library/general-documents). The already described variants (pathogenic, non-pathogenic and uncertain) were assigned after reviewing information available in BIC (Breast Cancer Information Core, https://research.nhgri.nih.gov/projects/bic/), ClinVar (http://www.ncbi.nlm.nih.gov/clinvar/), BRCA Share (former Universal Mutation Database, http://umd.be/) 1000 genomes (http://www.internationalgenome.org/), Leiden Open Variation Database (http://www.lovd.nl/3.0/home) and published manuscripts. Novel variants not classified under pathogenic, were analyzed with two integrative platforms a) HCI Breast Cancer Genes Prior Probabilities (http://priors.hci.utah.edu/PRIORS/references.php); b) Variant Effect Predictor [41] (VEP, http://www.ensembl.org/info/docs/tools/vep/index.html); and the recommended *in silico* tools [40] (ACMG and ENIGMA), c) Align-GVGD [42] (http://agvgd.hci.utah.edu), d) SIFT [43] (http://sift.jcvi.org), e) PROVEAN [44] (http://provean.jcvi.org/index.php), f) PolyPhen-2 [45] (http://genetics.bwh.harvard.edu/pph2). In addition, synonymous variants were also assessed for splicing effect using Human Splice Finder [46] (HSP, http://www.umd.be/HSF3/). Default parameters were applied for each tool.

### Founder haplotype determination for recurrent mutations

We analyzed a total of 11 Short Tandem Repeats (STR) markers. For mutations in *BRCA1*: D17S1323, D17S1322, D17S855, D17S1320, D17S1326 and D17S1327, covering 0.68 Mb, and for mutations in *BRCA2*: D13S260, D13S1698, D13S267, D13S171 and D13S310 covering 1.82 Mb. All loci were amplified by PCR using primers, end-labeled with different fluorophores (VIC, 6-FAM, PET and NED). PCR products were analyzed by capillary electrophoresis in an ABI PRISM 3130x fragment analyzer (Applied Technologies) using Peak Scanner Software 2 to confirm product amplification and size. The STR alleles associated to each recurrent mutation were grouped in haplotypes for all samples carrying the same mutation, and phases were confirmed by PHASE v2.1 [47, 48] (stephenslab.ucicago.edu/phase). A minimum common haplotype was determined to define a common ancestor.

## SUPPLEMENTARY MATERIALS TABLES





## References

[1] Miki Y, Swensen J, Shattuck-Eidens D, Futreal PA, Harshman K, Tavtigian S, Liu Q, Cochran C, Bennett LM, Ding W (1994). A strong candidate for the breast and ovarian cancer susceptibility gene BRCA1. Science.

[2] Wooster R, Bignell G, Lancaster J, Swift S, Seal S, Mangion J, Collins N, Gregory S, Gumbs C, Micklem G, Barfoot R, Hamoudi R, Patel S (1995). Identification of the breast cancer susceptibility gene BRCA2. Nature.

[3] Fackenthal JD, Olopade OI (2007). Breast cancer risk associated with BRCA1 and BRCA2 in diverse populations. Nat Rev Cancer.

[4] Jara L, Ampuero S, Santibáñez E, Seccia L, Rodríguez J, Bustamante M, Martínez V, Catenaccio A, Lay-Son G, Blanco R, Reyes JM (2006). BRCA1 and BRCA2 mutations in a South American population. Cancer Genet Cytogenet.

[5] Gallardo M, Silva A, Rubio L, Alvarez C, Torrealba C, Salinas M, Tapia T, Faundez P, Palma L, Riccio ME, Paredes H, Rodriguez M, Cruz A (2006). Incidence of BRCA1 and BRCA2 mutations in 54 Chilean families with breast/ovarian cancer, genotype-phenotype correlations. Breast Cancer Res Treat.

[6] Ruiz-Flores P, Sinilnikova OM, Badzioch M, Calderon-Garcidueñas AL, Chopin S, Fabrice O, González-Guerrero JF, Szabo C, Lenoir G, Goldgar DE, Barrera-Saldaña HA (2002). BRCA1 and BRCA2 mutation analysis of early-onset and familial breast cancer cases in Mexico. Hum Mutat.

[7] Torres D, Rashid MU, Gil F, Umana A, Ramelli G, Robledo JF, Tawil M, Torregrosa L, Briceno I, Hamann U (2007). High proportion of BRCA1/2 founder mutations in Hispanic breast/ovarian cancer families from Colombia. Breast Cancer Res Treat.

[8] Gomes MC, Costa MM, Borojevic R, Monteiro AN, Vieira R, Koifman S, Koifman RJ, Li S, Royer R, Zhang S, Narod SA (2007). Prevalence of BRCA1 and BRCA2 mutations in breast cancer patients from Brazil. Breast Cancer Res Treat.

[9] Solano AR, Aceto GM, Delettieres D, Veschi S, Neuman MI, Alonso E, Chialina S, Chacón RD, Renato MC, Podestá EJ (2012). BRCA1 and BRCA2 analysis of Argentinean breast/ovarian cancer patients selected for age and family history highlights a role for novel mutations of putative south-American origin. Springerplus.

[10] Delgado L, Fernández G, Grotiuz G, Cataldi S, González A, Lluveras N, Heguaburu M, Fresco R, Lens D, Sabini G, Muse IM (2011). BRCA1 and BRCA2 germline mutations in Uruguayan breast and breast-ovarian cancer families. Identification of novel mutations and unclassified variants. Breast Cancer Res Treat.

[11] Lara K, Consigliere N, Pérez J, Porco A (2012). BRCA1 and BRCA2 mutations in breast cancer patients from Venezuela. Biol Res.

[12] Levy-Lahad E, Catane R, Eisenberg S, Kaufman B, Hornreich G, Lishinsky E, Shohat M, Weber BL, Beller U, Lahad A, Halle D (1997). Founder BRCA1 and BRCA2 mutations in Ashkenazi Jews in Israel: frequency and differential penetrance in ovarian cancer and in breast-ovarian cancer families. Am J Hum Genet.

[13] Johannesdottir G, Gudmundsson J, Bergthorsson JT, Arason A, Agnarsson BA, Eiriksdottir G, Johannsson OT, Borg A, Ingvarsson S, Easton DF, Egilsson V, Barkardottir RB (1996). High prevalence of the 999del5 mutation in icelandic breast and ovarian cancer patients. Cancer Res.

[14] Thorlacius S, Olafsdottir G, Tryggvadottir L, Neuhausen S, Jonasson JG, Tavtigian SV, Tulinius H, Ogmundsdottir HM, Eyfjörd JE (1996). A single BRCA2 mutation in male and female breast cancer families from Iceland with varied cancer phenotypes. Nat Genet.

[15] Thorlacius S, Sigurdsson S, Bjarnadottir H, Olafsdottir G, Jonasson JG, Tryggvadottir L, Tulinius H, Eyfjörd JE (1997). Study of a single BRCA2 mutation with high carrier frequency in a small population. Am J Hum Genet.

[16] Górski B, Jakubowska A, Huzarski T, Byrski T, Gronwald J, Grzybowska E, Mackiewicz A, Stawicka M, Bebenek M, Sorokin D, Fiszer-Maliszewska Ł Haus O, Janiszewska H (2004). A high proportion of founder BRCA1 mutations in Polish breast cancer families. Int J Cancer.

[17] Krajc M, Teugels E, Zgajnar J, Goelen G, Besic N, Novakovic S, Hocevar M, De Grève J (2008). Five recurrent BRCA1/2 mutations are responsible for cancer predisposition in the majority of Slovenian breast cancer families. BMC Med Genet.

[18] Ferla R, Calò V, Cascio S, Rinaldi G, Badalamenti G, Carreca I, Surmacz E, Colucci G, Bazan V, Russo A (2007 (Suppl 6)). Founder mutations in BRCA1 and BRCA2 genes. Ann Oncol.

[19] Ashton-Prolla P, Vargas FR (2014). Prevalence and impact of founder mutations in hereditary breast cancer in Latin America. Genet Mol Biol.

[20] Ossa CA, Torres D (2016). Founder and Recurrent Mutations in BRCA1 and BRCA2 Genes in Latin American Countries: State of the Art and Literature Review. Oncologist.

[21] Gonzalez-Hormazabal P, Gutierrez-Enriquez S, Gaete D, Reyes JM, Peralta O, Waugh E, Gomez F, Margarit S, Bravo T, Blanco R, Diez O, Jara L (2011). Spectrum of BRCA1/2 point mutations and genomic rearrangements in high-risk breast/ovarian cancer Chilean families. Breast Cancer Res Treat.

[22] Farrugia DJ, Agarwal MK, Pankratz VS, Deffenbaugh AM, Pruss D, Frye C, Wadum L, Johnson K, Mentlick J, Tavtigian SV, Goldgar DE, Couch FJ (2008). Functional assays for classification of BRCA2 variants of uncertain significance. Cancer Res.

[23] Vega A, Campos B, Bressac-De-Paillerets B, Bond PM, Janin N, Douglas FS, Domènech M, Baena M, Pericay C, Alonso C, Carracedo A, Baiget M, Diez O (2001). The R71G BRCA1 is a founder Spanish mutation and leads to aberrant splicing of the transcript. Hum Mutat.

[24] Solano AR, Cardoso FC, Romano V, Perazzo F, Bas C, Recondo G, Santillan FB, Gonzalez E, Abalo E, Viniegra M, Michel JD, Nuñez LM, Noblia CM (2017). Spectrum of BRCA1/2 variants in 940 patients from Argentina including novel, deleterious and recurrent germline mutations: impact on healthcare and clinical practice. Oncotarget.

[25] Weitzel JN, Lagos V, Blazer KR, Nelson R, Ricker C, Herzog J, McGuire C, Neuhausen S (2005). Prevalence of BRCA mutations and founder effect in high-risk Hispanic families. Cancer Epidemiol Biomarkers Prev.

[26] Torres D, Bermejo JL, Rashid MU, Briceño I, Gil F, Beltran A, Ariza V, Hamann U (2017). Prevalence and Penetrance of BRCA1 and BRCA2 Germline Mutations in Colombian Breast Cancer Patients. Sci Rep.

[27] Dutil J, Golubeva VA, Pacheco-Torres AL, Diaz-Zabala HJ, Matta JL, Monteiro AN (2015). The spectrum of BRCA1 and BRCA2 alleles in Latin America and the Caribbean: a clinical perspective. Breast Cancer Res Treat.

[28] Weitzel JN, Clague J, Martir-Negron A, Ogaz R, Herzog J, Ricker C, Jungbluth C, Cina C, Duncan P, Unzeitig G, Saldivar JS, Beattie M, Feldman N (2013). Prevalence and type of BRCA mutations in Hispanics undergoing genetic cancer risk assessment in the southwestern United States: a report from the Clinical Cancer Genetics Community Research Network. J Clin Oncol.

[29] Hernández JE, Llacuachaqui M, Palacio GV, Figueroa JD, Madrid J, Lema M, Royer R, Li S, Larson G, Weitzel JN, Narod SA (2014). Prevalence of BRCA1 and BRCA2 mutations in unselected breast cancer patients from medellín, Colombia. Hered Cancer Clin Pract.

[30] Villarreal-Garza C, Alvarez-Gómez RM, Pérez-Plasencia C, Herrera LA, Herzog J, Castillo D, Mohar A, Castro C, Gallardo LN, Gallardo D, Santibáñez M, Blazer KR, Weitzel JN (2015). Significant clinical impact of recurrent BRCA1 and BRCA2 mutations in Mexico. Cancer.

[31] Abugattas J, Llacuachaqui M, Allende YS, Velásquez AA, Velarde R, Cotrina J, Garcés M, León M, Calderón G, de la Cruz M, Mora P, Royer R, Herzog J (2015). Prevalence of BRCA1 and BRCA2 mutations in unselected breast cancer patients from Peru. Clin Genet.

[32] Alemar B, Herzog J, Brinckmann Oliveira Netto C, Artigalás O, Schwartz IV, Matzenbacher Bittar C, Ashton-Prolla P, Weitzel JN (2016). Prevalence of Hispanic BRCA1 and BRCA2 mutations among hereditary breast and ovarian cancer patients from Brazil reveals differences among Latin American populations. Cancer Genet.

[33] Felix GE, Abe-Sandes C, Machado-Lopes TM, Bomfim TF, Guindalini RS, Santos VC, Meyer L, Oliveira PC, Cláudio Neiva J, Meyer R, Romeo M, Betânia Toralles M, Nascimento I, Abe-Sandes K (2014). Germline mutations in BRCA1, BRCA2, CHEK2 and TP53 in patients at high-risk for HBOC: characterizing a Northeast Brazilian Population. Hum Genome Var.

[34] Infante M, Durán M, Esteban-Cardeñosa E, Miner C, Velasco E (2006). High proportion of novel mutations of BRCA1 and BRCA2 in breast/ovarian cancer patients from Castilla-León (central Spain). J Hum Genet.

[35] Miramar MD, Calvo MT, Rodriguez A, Antón A, Lorente F, Barrio E, Herrero A, Burriel J (2008). García de Jalón A. Genetic analysis of BRCA1 and BRCA2 in breast/ovarian cancer families from Aragon (Spain): two novel truncating mutations and a large genomic deletion in BRCA1. Breast Cancer Res Treat.

[36] Infante M, Durán M, Acedo A, Pérez-Cabornero L, Sanz DJ, García-González M, Beristain E, Esteban-Cardeñosa E, de la Hoya M, Teulé A, Vega A, Tejada MI, Lastra E (2010). BRCA1 5272-1G>A and BRCA2 5374delTATG are founder mutations of high relevance for genetic counselling in breast/ovarian cancer families of Spanish origin. Clin Genet.

[37] Zúñiga JP

[38] Rocco P, Morales C, Moraga M, Miquel JF, Nervi F, Llop E, Carvallo P, Rothhammer F (2002). [Genetic composition of the Chilean population. Analysis of mitochondrial DNA polymorphism]. [Article in Spanish]. Rev Med Chil.

[39] Lahiri DK, Nurnberger JI (1991). A rapid non-enzymatic method for the preparation of HMW DNA from blood for RFLP studies. Nucleic Acids Res.

[40] Richards CS, Bale S, Bellissimo DB, Das S, Grody WW, Hegde MR, Lyon E, Ward BE, Molecular Subcommittee of the ACMG Laboratory Quality Assurance Committee (2008). ACMG recommendations for standards for interpretation and reporting of sequence variations: Revisions 2007. Genet Med.

[41] McLaren W, Pritchard B, Rios D, Chen Y, Flicek P, Cunningham F (2010). Deriving the consequences of genomic variants with the Ensembl API and SNP Effect Predictor. Bioinformatics.

[42] Tavtigian SV, Deffenbaugh AM, Yin L, Judkins T, Scholl T, Samollow PB, de Silva D, Zharkikh A, Thomas A (2006). Comprehensive statistical study of 452 BRCA1 missense substitutions with classification of eight recurrent substitutions as neutral. J Med Genet.

[43] Ng PC, Henikoff S (2001). Predicting deleterious amino acid substitutions. Genome Res.

[44] Choi Y, Sims GE, Murphy S, Miller JR, Chan AP (2012). Predicting the functional effect of amino acid substitutions and indels. PLoS One.

[45] Adzhubei IA, Schmidt S, Peshkin L, Ramensky VE, Gerasimova A, Bork P, Kondrashov AS, Sunyaev SR (2010). A method and server for predicting damaging missense mutations. Nat Methods.

[46] Desmet FO, Hamroun D, Lalande M, Collod-Béroud G, Claustres M, Béroud C (2009). Human Splicing Finder: an online bioinformatics tool to predict splicing signals. Nucleic Acids Res.

[47] Stephens M, Smith NJ, Donnelly P (2001). A new statistical method for haplotype reconstruction from population data. Am J Hum Genet.

[48] Stephens M, Donnelly P (2003). A comparison of bayesian methods for haplotype reconstruction from population genotype data. Am J Hum Genet.

