# Supplementary material for: BRCA1 and BRCA2 founder mutations account for 78% of germline carriers among hereditary breast cancer families in Chile
Source: Oncotarget. 2017 Jun 29;8(43):74233–43. doi: 10.18632/oncotarget.18815 (PMC5650336; doi:10.18632/oncotarget.18815)
Supplement: Supplementary file 1 [file oncotarget-08-74233-s001.pdf]

BRCA1 and *BRCA2* founder mutations account for 78% of germline carriers among hereditary breast cancer families in Chile

Supplementary Materials

Supplementary Table 1: Previously reported rare variants in *BRCA1* and *BRCA2*, found in Chilean breast cancer patients. See Supplementary_Table_1
