# Supplementary material for: BRCA1 and BRCA2 founder mutations account for 78% of germline carriers among hereditary breast cancer families in Chile
Source: Oncotarget. 2017 Jun 29;8(43):74233–43. doi: 10.18632/oncotarget.18815 (PMC5650336; doi:10.18632/oncotarget.18815)
Supplement: Supplementary file 2 [file oncotarget-08-74233-s002.docx]

**Supplementary Table 1: Previously reported rare variants in *BRCA1* and *BRCA2*, found in Chilean breast cancer patients**

| **Gene** | **Exon/Intron** | **HGVS Nomenclature** | **Protein Effect** | **Frequency in breast cancer patients (%) this study** | **Rs Number** | **BIC Clinical significance** | **ClinVar Clinical significance** | **BRCA Share Clinical significance** | **Functional assay** | **Reported co-occurrence with a BRCA1/2 pathogenic variant** | **Conclusion** |
| --- | --- | --- | --- | --- | --- | --- | --- | --- | --- | --- | --- |
|  |  |  |  |  |  |  |  |  |  |  |  |
| **SYNONIMOUS VARIANTS** |  |  |  |  |  |  |  |  |  |  |  |
| *BRCA1* | 9 | c.591C>T | p.Cys197= | 0,3 | rs1799965 | Not pathogenic | Benign/likely benign | Neutral | Dosil et al 2010, Houdayer et al 2012 | Yes | Not pathogenic |
|  | 11 | c.981A>G | p.Thr327= | 0,3 | rs1800063 | Unknown | Benign/likely benign | Neutral | - | Yes | Not pathogenic |
|  | 11 | c.1071A>G | p.Lys357= | 1,6 | rs786202159 | - | Likely benign | - | - | Not informed | Likely not pathogenic |
|  | 11 | c.2733A>G | p.Gly911= | 0,3 | rs1800740 | Unknown | Benign/likely benign | Neutral | - | Yes | Not pathogenic |
|  | 11 | c.3238T>C | p.Leu1080= | 0,3 | rs754597283 | - | Likely benign | Unclassified Variant | - | Not informed | Likely not pathogenic |
| *BRCA2* | 3 | c.222G>C | p.Leu74= | 0,3 | rs863224303 | - | Likely benign | - | - | Not informed | Likely not pathogenic |
|  | 10 | c.1371G>A | p.Lys457= | 0,3 | rs786202479 | - | Likely benign | - | - | Not informed | Likely not pathogenic |
|  | 11 | c.1938C>T | p.Ser646= | 0,7 | rs28897711 | Not pathogenic | Benign/likely benign | Neutral | - | Yes | Not pathogenic |
|  | 11 | c.4686A>G | p.Gln1562= | 0,3 | rs28897730 | - | Benign/likely benign | Likely neutral | - | Yes | Not pathogenic |
|  |  |  |  |  |  |  |  |  |  |  |  |
| **NON-SYNONIMOUS VARIANTS** |  |  |  |  |  |  |  |  |  |  |  |
| *BRCA1* | 11 | c.2312T>C | p.Leu771Ser | 0,3 | rs730881481 | - | Uncertain | Unclassified Variant | - | not informed | Uncertain |
|  | 11 | c.2662C>T | p.His888Tyr | 0,3 | rs80357480 | Unknown | Uncertain | Unclassified Variant | - | Yes | Uncertain |
|  | 11 | c.3024G>A | p.Met1008Ile | 0,3 | rs1800704 | Unknown | Benign | Neutral | Bouwman et al 2013 | Yes | Not pathogenic |
|  | 15 | c.4535G>T | p.Ser1512Ile | 0,7 | rs1800744 | Not pathogenic | Benign | Neutral | Phelan et al 2005, Caligo et al 2008 | Yes | Not pathogenic |
|  | 16 | c.4766G>A | p.Arg1589His | 0,3 | rs80357341 | Unknown | Likely benign/ uncertain | Unclassified Variant | Quiles et al 2013 | Yes | Not pathogenic |
| *BRCA2* | 10 | c.1889 C>T | p.Thr630Ile | 0,3 | rs80358479 | Unknown | Benign | Likely neutral | - | Yes | Not pathogenic |
|  | 11 | c.2389A>C | p.Lys797Gln | 0,3 | rs587782737 | - | Uncertain | - | - | not informed | Uncertain |
|  | 11 | c.4535G>C | p.Arg1512Pro | 0,3 | rs80358685 | Unknown | Uncertain | Unclassified Variant | - | not informed | Uncertain |
|  | 11 | c.6290C>T | p.Thr2097Met | 0,3 | rs80358866 | Unknown | Benign/likely benign | Unclassified Variant | - | not informed | Uncertain |
|  | 11 | c.6443C>A | p.Ser2148Tyr | 0,3 | rs80358880 | Unknown | Benign/likely benign/uncertain | Unclassified Variant | - | not informed | Uncertain |
|  | 15 | c.7507G>A | p.Val2503Ile | 0,3 | rs587782191 | - | Uncertain | Unclassified Variant | - | not informed | Uncertain |
|  | 18 | c.8149G>T | p.Ala2717Ser | 0,3 | rs28897747 | Not pathogenic | Benign | Neutral | - | Yes | Not pathogenic |
|  | 18 | c.8153T>C | p.Ile2718Thr | 1,0 | rs80359060 | Unknown | Uncertain | Unclassified Variant | - | not informed | Uncertain |
|  | 20 | c.8545A>G | p.Lys2849Glu | 0,3 | rs80359109 | Unknown | Uncertain | Unclassified Variant | - | not informed | Uncertain |
|  | 22 | c.8850G>T | p.Lys2950Asn | 0,3 | rs28897754 | Unknown | Benign/likely benign | Neutral | Houdayer 2012 | Yes | Not pathogenic |
|  | 23 | c.9104A>C | p.Tyr3035Ser | 0,3 | rs80359165 | Unknown | Benign/likely benign | Unclassified Variant | Thery et al 2011 | Yes | Not pathogenic |
|  | 25 | c.9292T>C | p.Tyr3098His | 0,7 | rs41293521 | Unknown | Benign | Likely neutral | - | Yes | Not pathogenic |
|  |  |  |  |  |  |  |  |  |  |  |  |
| **NON-CODING VARIANTS** |  |  |  |  |  |  |  |  |  |  |  |
| *BRCA1* | I2 | c.81-55T>C | NA | 0,3 | - | - | - | Likely neutral | - | Not informed | Likely not pathogenic |
|  | I13 | c.4358-10C>T | NA | 0,3 | rs80358111 | Not pathogenic | Benign/likely benign | Neutral | Houdayer et al 2012 | Yes | Not pathogenic |
|  | I14 | c.4485-10 A>G | NA | 0,3 | rs863224420 | - | Likely benign | - | - | Not informed | Likely not pathogenic |
| *BRCA2* | I11 | c.6841+92T>C | NA | 0,7 | - | - | - | Unclassified Variant | - | Not informed | Uncertain |
|  | I17 | c.7976+23C>T | NA | 0,3 | rs183623188 | Unknown | Likely benign | Likely neutral | Houdayer et al 2012 | Yes | Not pathogenic |
|  | I17 | c.7976+57G>C | NA | 0,3 | - | - | - | Unclassified Variant | Ruiz de Garibay et al 2014 | Not informed | Not pathogenic |
|  | I25 | c.9501+9A>C | NA | 0,3 | rs81002867 | Unknown | Benign/likely benign | Neutral | Campos et al 2003 | Yes | Not pathogenic |
|  |  |  |  |  |  |  |  |  |  |  |  |
| GeneBank Accession Numbers: *BRCA1:* L78833 and *BRCA2:* AY436640 | |  |  |  |  |  |  |  |  |  |  |
